# Adjusting Cholera Models to Recent Experimental Data

**DOI:** 10.1371/journal.pmed.0030028

**Published:** 2005-12-06

Infectious disease modeling has a long history, going back to at least Daniel Bernouli's smallpox model from 1760. The discipline is driven by the desire to understand the dynamics of an outbreak or epidemic in order to plan control strategies. The hope is that better models will also allow prediction of future outbreaks, and inform not just preparedness but also prevention strategies. One critical component of all infectious disease models—and by some experts seen as the bottleneck of most models—is the mode of transmission. Interaction between modelers and experimentalists who study transmission from environment to humans and transmission between humans is therefore crucial.

In 2002, Andrew Camilli and colleagues reported that passage through a human host potentiated the infectivity of the cholera pathogen Vibrio cholerae (Nature 417: 642–645). They isolated and characterized V. cholerae from human stools, and found that passage through the human gastrointestinal tract induces a transient hyperinfectious state in the bacteria, which is perpetuated for a number of hours, even outside the human host. (Infectiousness was determined in competition assays in infant mice.) This hyperinfectious state was associated with specific gene expression profiles that differed from those of bacteria in their normal aquatic reservoirs. [Fig pmed-0030028-g001]

**Figure pmed-0030028-g001:**
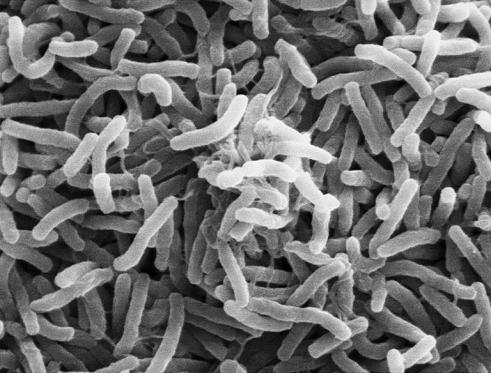
Scanning electron micropgaph of Vibrio cholerae

The study caught the attention of David Hartley and colleagues, who saw a chance to improve modeling in the field of cholera epidemics. Hartley was interested because Camilli's results shed new light on a fundamental question in cholera epidemiology, namely, what is the relative importance of human-to-human (i.e., fecal to oral) versus environment-to-human infection (through contaminated food or water)? If the infective dose of bacteria that have become hyperinfectious because of recent passage through a human host is lower than that of environmental V. cholerae, this would support a crucial role of human-to-human transmission in the generation of cholera epidemics.

Hartley and colleagues found that incorporation of the existence of a hyperinfectious state into their disease models resulted in a much better fit of the predictions with the observed explosive epidemic patterns of past cholera outbreaks. On one hand, this result lends theoretical support for Camilli's results and suggests that his findings in laboratory animals have clinical relevance. On the other hand, it strongly suggests that human-to-human transmission is crucial for cholera epidemics and pandemics, and that health measures must focus on minimizing risk of transmission of the short-lived hyperinfectious form of V. cholerae that results from transmission through a human host. Finally, there is the intriguing possibility that similar hyperinfectious states exist for other bacteria, something that seems well worth exploring.

